# High‐risk group of upper and middle mediastinal lymph node metastasis in patients with esophagogastric junction carcinoma

**DOI:** 10.1002/ags3.12215

**Published:** 2018-10-13

**Authors:** Masahiro Yura, Hiroya Takeuchi, Kazumasa Fukuda, Rieko Nakamura, Koichi Suda, Norihito Wada, Hirofumi Kawakubo, Yuko Kitagawa

**Affiliations:** ^1^ Department of Surgery Keio University School of Medicine Tokyo Japan; ^2^ Department of Surgery Hamamatsu University School of Medicine Hamamatsu‐shi Japan

**Keywords:** esophagogastric junction carcinoma, length of esophageal invasion, mediastinal lymph node metastasis, treatment algorithm, tumor epicenter

## Abstract

**Aim:**

The aim of the present study was to clarify esophagogastric junction (EGJ) carcinoma patients who are at high risk of upper and middle mediastinal lymph node (MLN) metastasis.

**Methods:**

This was a retrospective study and included 110 consecutive patients with EGJ carcinoma who underwent R0/R1 resection at Keio University Hospital between January 2000 and December 2013.

**Results:**

Of the 110 patients, 18 (16.3%) had MLN metastasis, and the number increased to 23 (20.9%) when recurrence cases were added (adenocarcinoma, N = 11; squamous cell carcinoma, N = 12). Patients whose tumor epicenter was located above the EGJ had a significantly higher incidence of MLN metastasis/recurrence (18/51 [35.3%]) than those whose tumor epicenter was located below the EGJ (5/59 [8.5%]). The MLN metastasis/recurrence rate was particularly high when the distance from the EGJ to the proximal edge of the primary tumor was >3 cm for the upper and middle mediastinum (18.8%). Patients in a selected group (≥T2 and tumor epicenter located above the EGJ or below the EGJ with ≥3 cm esophageal invasion) showed 17.9% and 15.4% upper and middle MLN metastasis/recurrence rates, respectively. Therapeutic value of MLN dissection was relatively high (#105 + 106: 8.9, #110: 12.2).

**Conclusions:**

Therapeutic value of MLN dissection to treat EGJ carcinomas was relatively high in patients with MLN metastasis. Our algorithm could select patients at high risk for MLN metastasis.

## 1 INTRODUCTION

The incidence of esophagogastric junction (EGJ) carcinoma has increased around the world,^1^, ^2^, ^3^ including Japan. However, the optimal surgical treatment for EGJ carcinoma remains controversial.^4^, ^5^, ^6^, ^7^, ^8^, ^9^, ^10^, ^11^, ^12^, ^13^ In Western countries, adenocarcinoma (AC) is the major histological type, and the Siewert classification has been adopted.^14^ The surgical approach for EGJ adenocarcinoma is based on the tumor location. Usually, a transthoracic approach is carried out for patients with Siewert type I tumors, whereas the transhiatal approach is used for those with Siewert type III tumors. Both approaches have been used for Siewert type II tumors. A Dutch trial compared the right thoracic and transhiatal approaches for Siewert tumor types I and II.^4^ Although the survival rate of patients with Siewert type II tumors was not different between the groups, a subgroup analysis of nodal metastatic patients showed that survival in patients who underwent the transthoracic approach was significantly better than that of patients who underwent the transhiatal approach.^4^ Squamous cell carcinoma (SCC) of the EGJ is often observed in Asian populations. Therefore, Nishi's classification of EGJ carcinoma has been used in Japan.^15^ This definition describes EGJ carcinoma as a tumor whose tumor epicenter is located within a 2‐cm area above and below the EGJ, regardless of histological type. Although this classification includes both histological types, most patients with AC have received a resection of the lower esophagus and stomach, whereas those with SCC have undergone subtotal esophagectomy, reflecting differences in the dominant tumor location.

Siewert et al^16^ showed that R0 resection was a prognostic factor in patients with EGJ carcinoma. Thus, appropriate prophylactic mediastinal lymph node (MLN) dissection is necessary for EGJ carcinoma. We usually use two major surgical approaches for EGJ carcinoma to retrieve MLN: the transthoracic approach and the transhiatal approach. The transthoracic approach is a more invasive procedure, which may increase morbidity and worsen the patient's quality of life after surgery. Although lymph nodes in the lower mediastinal region can be retrieved using both approaches, lymph node dissection of the upper‐middle mediastinum can only be carried out with the transthoracic approach. However, the optimal surgical approach for patients with EGJ carcinoma remains unclear. In the present study, we aimed to explore high‐risk EGJ carcinoma patients who have upper and middle MLN metastasis/recurrence and underwent the transthoracic approach. We retrospectively collected the medical records of patients with EGJ carcinoma (diagnosed according to Nishi's classification^15^) who underwent surgical resection.

## 2 MATERIALS AND METHODS

### 2.1 Patients

This was a retrospective study and included 110 consecutive patients diagnosed with EGJ carcinoma who subsequently underwent curative surgical resection at Keio University Hospital (Tokyo, Japan) between January 2000 and December 2013. In an additional study, 22 EGJ carcinoma patients who were treated with surgical resection between January 2014 and November 2016 were included to prove the suitability of our algorithm, which was created according to the results of our retrospective study. EGJ carcinoma was defined according to Nishi's classification.^15^ EGJ was identified from the resected specimen and was defined using the level of macroscopic caliber change. Tumors with epicenters located in the area of the EGJ, extending from 2 cm above to 2 cm below the EGJ, were designated as EGJ carcinomas.

### 2.2 Follow up

For postoperative follow up, we usually carry out computed tomography (CT) scan every 6 months and endoscopic examination every year after operation.

### 2.3 Evaluation and lymph node station

The 7th edition of the Union for International Cancer Control tumor‐node‐metastasis classification of esophageal cancer was used for tumor staging,^17^ and the Japanese classification of esophageal carcinoma was used to number the lymph node stations.^15^ The upper mediastinal area included the upper thoracic paraesophageal (#105) and thoracic paratracheal lymph nodes (#106). The middle mediastinal area included the subcarinal (#107), middle thoracic paraesophageal (#108), and main bronchus lymph nodes (#109). The lower mediastinal area included the lower thoracic paraesophageal (#110), supradiaphragmatic (#111), and posterior MLN (#112).

### 2.4 Therapeutic value of lymph node dissection

To evaluate the therapeutic value of dissecting each lymph node station, we used the modified method presented in 1995 by Sasako et al^18^ who used the therapeutic value index. The therapeutic value of nodal dissection was based on multiplication of the lymph node metastasis rate and the 3‐year survival rate in patients with lymph node metastases (as a percentage). The rate of lymph node metastasis was calculated by multiplying the number of patients with lymph node metastases for each station and the number of those in whom that station was retrieved. The 3‐year overall survival rates in patients with lymph node metastasis were calculated for each nodal station, regardless of lymph node metastasis for other stations.

### 2.5 Statistical analysis

Statistical analysis was carried out using SPSS statistical software (ver. 23; SPSS Inc., Chicago, IL, USA). Clinical and pathological variables were analyzed using Pearson's chi‐squared and Mann‐Whitney *U*‐tests. Multivariate logistic regression analysis was carried out to identify the risk factors for MLN metastasis. *P*‐value <0.05 was considered significant.

### 2.6 Decision‐making for operative procedures

Before 2014, when a surgical algorithm for EGJ carcinoma was published in the Japanese Gastric Cancer Guideline 4th edition,^19^ clear algorithms in guidelines were lacking and surgery was carried out at the discretion of the operator or based on decisions made at a conference. Subtotal esophagectomy was the first choice of treatment for EGJ SCC as lower esophageal carcinomas. However, depending on the general condition of the patient, lower esophageal resection by a transhiatal approach was also carried out. Before 2004, the left transthoracic approach was selected when mediastinal anastomosis using the transhiatal approach was thought to be difficult. For AC, when CT showed mediastinal lymphadenopathy, lymph node dissection and esophagectomy were both carried out. The transhiatal approach was used in cases lacking indications for upper and/or middle MLN metastasis. However, even in such cases, a transthoracic approach was preferred when the anastomotic position was high and transhiatal anastomosis was expected to be difficult. After the guidelines were published, the procedures were basically determined according to the guidelines.

## 3 RESULTS

### 3.1 Background characteristics and pathological findings of the patients

**Figure 1:**
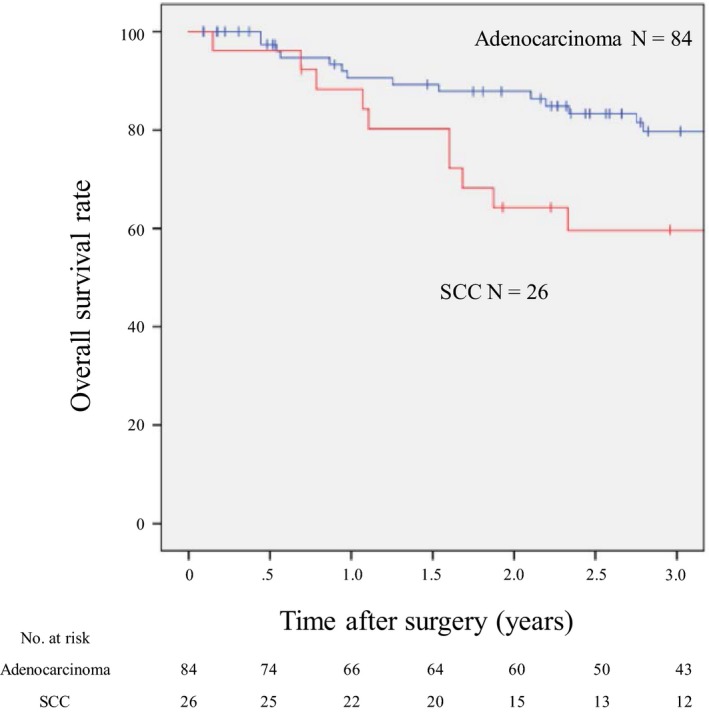
Survival curve for all patients with adenocarcinoma (AC) and squamous cell carcinoma (SCC). The 3‐y survival rate was 79.7% in patients with AC and 59.6% in patients with SCC

Background characteristics and pathological findings of the patients are presented in Table 1. A total of 110 patients were enrolled, consisting of 84 with AC and 26 with SCC. Although no significant differences were identified in terms of age, gender, or adjuvant therapy between the two groups, the surgical procedures and neoadjuvant therapy were found to vary. The transhiatal approach was mainly carried out in AC patients (80.9%), whereas only 11.5% of patients with SCC underwent this procedure. Subtotal esophagectomy was primarily used in patients with SCC (80.8%), as compared to 14.3% of patients with AC. Total gastrectomy (40.4%) and proximal gastrectomy (39.2%) were mainly used in AC patients, whereas partial gastrectomy for making a gastric tube was common in patients with SCC (80.8%). Length from tumor center to EGJ was significantly different between the AC and the SCC groups (AC: 5.7 ± 9.5 mm vs SCC: –9.9 ± 8.7 mm; *P* < 0.001). Patients with SCC had significantly higher tumor center locations and oral tumor borders, as compared to AC patients (*P *<* *0.001). Advanced cases (≥pT2) were noted in 61.9% (52/84) of patients in the AC group, and in 80.8% (21/26) of patients in the SCC group. LN metastasis was observed in 45.2% (38/84) of AC patients and in 76.9% (20/26) of SCC patients. We calculated the 3‐year overall survival rates according to histological type. Median follow‐up period was 51.5 months with a range of 1‐179 months. The 3‐year overall survival rate was 79.7% in patients with AC and 59.6% in patients with SCC (Figure 1). There was no significant difference in overall survival between patients with AC and SCC (*P *=* *0.222).

**Table 1:** Background characteristics and pathological findings of the patients

	All patients N = 110	Adenocarcinoma N = 84	SCC N = 26	*P*‐value
Age (y, mean ± SD)	65.9 ± 11.6	65.6 ± 12.6	67.0 ± 7.3	0.470
Gender				
Male	87	67	20	0.756
Female	23	17	6	
Surgical procedure				
Approach (THA/RTA/LTA)	71/35/4	68/14/2	3/21/2	<0.001
Esophagectomy (subtotal/lower)	33/77	12/72	21/5	<0.001
Gastrectomy				
[Total/proximal/partial (gastric tube)]	38/34/38	34/33/17	4/1/21	<0.001
Splenectomy (±)	18/92	17/67	1/25	0.038
Neoadjuvant therapy (±)	25/85	12/72	13/13	<0.001
Adjuvant therapy (±)	25/85	20/64	5/21	0.626
Tumor diameter (mm), mean ± SD	38.6 ± 19.4	37.7 ± 20.3	41.6 ± 16.2	0.370
Location of tumor center from the EGJ (mm), mean ± SD	2.1 ± 11.4	5.7 ± 9.5	−9.9 ± 8.7	<0.001
Location of proximal tumor border from the EGJ (mm), mean ± SD	−14.5 ± 15.4	−10.1 ± 13.5	−28.8 ± 12.5	<0.001
Location of distal tumor border from the EGJ (mm), mean ± SD	19.0 ± 14.6	21.7 ± 14.3	10.2 ± 12.1	<0.001
Postoperative diagnosis				
Pathological T stage				
pT1	37	32	5	0.026
pT2	16	16	0	
pT3	47	27	20	
pT4	10	9	1	
Pathological N stage				
pN0	52	46	6	0.008
pN1	28	18	10	
pN2	18	13	5	
pN3	12	7	5	
UICC stage				
pStage IA	31	30	1	0.003
pStage IB	11	11	0	
pStage IIA	10	5	5	
pStage IIB	7	5	2	
pStage IIIA	21	12	9	
pStage IIIB	12	9	3	
pStage IIIC	17	11	6	
pStage IV	1	1	0	

LTA, left thoracoabdominal approach; RTA, right thoracoabdominal approach; THA, abdominal‐transhiatal approach; UICC, Union for International Cancer Control.

### 3.2 Therapeutic value of lymph node dissection

Metastasis to MLN was detected in seven patients with AC (8.3%) and in eleven patients with SCC (42.3%). There were no patients who had lymph node metastasis to the pyloric region. The therapeutic value of extended nodal dissection of each lymph node station is shown in Table 2. High therapeutic values were observed in the lesser curvature areas, including stations 1, 3, and 7 (#1: 14.3, #3: 10.0, #7: 6.4). The number of patients who underwent lymph node dissection was limited (upper MLN, N = 30; lower MLN, N = 51), but the therapeutic index was relatively high for upper MLN (#105 + #106: 8.9) and lower MLN (#110: 12.2), as shown in Table 2.

**Table 2:** Therapeutic value of lymph node dissection

LN station	No. of patients with metastatic nodes	No. of patients in whom the station was dissected	Incidence of lymph node metastasis (%)	3‐y survival rate of patients with metastatic nodes (%)	Therapeutic index
102 + 104	0	8	0.0	0.0	0.0
105 + 106	5	30	16.7	53.3	8.9
107 + 108 + 109	4	35	11.4	0.0	0.0
111 + 112	4	49	8.2	25.0	2.1
110	10	51	19.6	62.2	12.2
1	30	110	27.3	52.4	14.3
2	23	110	20.9	36.7	7.7
3	26	110	23.6	42.5	10.0
4sa	1	76	1.3	100.0	1.3
4sb	0	73	0.0	0.0	0.0
4d	0	43	0.0	0.0	0.0
5	0	44	0.0	0.0	0.0
6	0	41	0.0	0.0	0.0
7	18	110	16.4	39.2	6.4
8a	2	73	2.7	0.0	0.0
9	4	73	5.5	50.0	2.8
10	2	24	8.3	50.0	4.2
11p	2	60	3.3	0.0	0.0
11d	0	27	0.0	0.0	0.0
12a	0	13	0.0	0.0	0.0

Cervical lymph node station numbers. 102 deep cervical lymph nodes (LN), 104 supraclavicular LN.

Thoracic lymph node station numbers. 105 upper thoracic paraesophageal LN, 106 recL left recurrent nerve LN, 106 recR right recurrent nerve LN, 108 middle thoracic paraesophageal LN, 109 main bronchus LN, 110 lower thoracic paraesophageal LN, 111 supradiaphragmatic LN.

Abdominal lymph node station numbers. 1 Right cardiac LN, 2 left cardiac LN, 3 LN along the lesser curvature, 4sa left greater curvature LN along the short gastric arteries, 4sb left greater curvature LN along the left gastroepiploic artery, 4d right greater curvature LN along the right gastroepiploic artery, 5 suprapyloric LN, 6 infrapyloric LN, 7 LN along the left gastric artery, 8a LN along the common hepatic artery anterosuperior group, 9 LN along the celiac artery, 10 LN at the splenic hilum, 11p LN along the proximal splenic artery, 11d LN along the distal splenic artery, 12a LN along the proper hepatic artery.

### 3.3 Recurrence patterns

Of all patients, 30.9% (34/110) developed recurrence, of whom 18 (16.4%) showed nodal recurrence in one or more regions (lymphatic recurrence: para‐aortic n = 10, gastric region n = 6, mediastinal region n = 9, cervical region n = 2; hematogenous recurrence: lung n = 7, liver n = 8, bone n = 3, peritoneum n = 6).

### 3.4 Mediastinal lymph node metastasis and recurrence

Mediastinal lymph node metastasis was observed in 18 patients, and the number increased to 23 when the recurrence cases were added (AC, N = 11; SCC, N = 12). Frequency of MLN metastasis or recurrence was higher in patients with SCC, as compared to those with AC (46.2% and 13.1%, respectively). Background characteristics and pathological findings of patients with and without MLN metastasis/recurrences are shown in Table 3. Of the 23 patients, rates for MLN metastasis were significantly higher when the tumor epicenter was located above the EGJ (18/51: 35.3%), as compared to when the epicenter was located below the EGJ (5/59: 8.5%). In these patients, two pT1 cases presented MLN metastasis, and the tumor epicenter was located above the EGJ in both cases. In contrast, five patients whose tumor epicenter was located below the EGJ and who had MLN metastasis/recurrence were diagnosed with advanced carcinoma (pT2/T3/T4: 1/3/1).

**Table 3:** Background characteristics and pathological findings of patients with and without mediastinal lymph node metastasis or recurrences

	Mediastinal lymph node metastasis or recurrences (+) N = 23	Mediastinal lymph node metastasis and recurrences (−) N = 87	*P*‐value
Histological type (adenocarcinoma/SCC)	11/12	73/14	0.001
Age (y, mean ± SD)	67.6 ± 9.4	65.5 ± 12.1	0.438
Gender			
Male	15	72	0.064
Female	8	15	
Surgical procedure			
Approach (THA/RTA/LTA)	6/16/1	65/19/3	<0.001
Esophagectomy (subtotal/lower)	16/7	17/70	<0.001
Gastrectomy			
[Total/proximal/partial (gastric tube)]	7/0/16	31/34/22	<0.001
Splenectomy (±)	3/20	15/72	0.451
Neoadjuvant therapy (±)	9/14	16/71	0.049
Adjuvant therapy (±)	8/15	17/70	0.121
Tumor diameter (mm), mean ± SD	43.8 ± 19.3	37.2 ± 19.3	0.438
Location of tumor center from the EGJ (mm), mean ± SD	−7.5 ± 10.9	4.6 ± 10.1	<0.001
Location of proximal tumor border from the EGJ (mm), mean ± SD	−27.0 ± 15.2	−11.2 ± 13.8	<0.001
Location of distal tumor border from the EGJ (mm), mean ± SD	12.9 ± 13.1	20.6 ± 14.6	0.024
Postoperative diagnosis			
Pathological T stage			
pT1	2	35	0.026
pT2	1	15	
pT3	17	30	
pT4	3	7	
Pathological N stage			
pN0	0	52	0.008
pN1	8	20	
pN2	8	10	
pN3	7	5	
UICC stage			
pStage IA	0	31	0.003
pStage IB	0	11	
pStage IIA	0	10	
pStage IIB	1	6	
pStage IIIA	8	13	
pStage IIIB	6	6	
pStage IIIC	8	9	
pStage IV	0	1	

LTA, left thoracoabdominal approach; RTA, right thoracoabdominal approach; THA, abdominal‐transhiatal approach; UICC, Union for International Cancer Control.

Of the total of 110 patients, 12 were found to have upper‐middle MLN metastasis/recurrence. In these patients, eight were AC and four were SCC, and they all had ≥pT2 tumor (pT2/T3/T4: 1/8/3). No significant difference was identified between the two histological types (Table 4). Among all patients, MLN metastasis and recurrence rates were found to be high when the distance from the EGJ to the proximal edge of the primary tumor was >3 cm for upper and middle mediastinum (18.8%) and >2 cm for lower mediastinum (15.0%) (Table 5).

**Table 4:** Clinical parameters and pathological findings of patients with upper and middle mediastinal lymph node metastasis and recurrence

	AC N = 8	SCC N = 4	*P*‐value
Tumor diameter (mm), mean ± SD	47.4 ± 22.7	46.2 ± 27.5	0.941
Location of tumor center from the EGJ (mm), mean ± SD	−1.3 ± −12.5	−14.5 ± 6.4	0.079
Location of proximal tumor border from the EGJ (mm), mean ± SD	−23.6 ± 16.9	−33.8 ± 17.1	0.353
No. of total lymph node metastasis	5.2 ± 3.1	5.5 ± 4.4	0.911
Postoperative diagnosis			
Pathological T stage			
pT1	0	0	0.759
pT2	1	0	
pT3	5	3	
pT4	2	1	
Pathological N stage			
pN0	0	0	0.786
pN1	2	1	
pN2	3	1	
pN3	3	2	
UICC stage			
pStage IA	0	0	0.360
pStage IB	0	0	
pStage IIA	0	0	
pStage IIB	1	0	
pStage IIIA	1	1	
pStage IIIB	3	0	
pStage IIIC	3	3	
pStage IV	0	0	

AC, adenocarcinoma; SCC, squamous cell carcinoma; UICC, Union for International Cancer Control.

**Table 5:** Metastasis or recurrence rates in the upper, middle, and lower mediastinal lymph nodes stratified by the distance from the EGJ to the proximal edge of primary tumor

Location of MLN	Location of proximal tumor border from the EGJ (mm)		
	0‐20 mm	21‐30 mm	30 mm<
Upper mediastinum	5/74 (6.8%)	2/20 (10.0%)	3/16 (18.8%)
Middle mediastinum	3/74 (4.1%)	1/20 (5.0%)	3/16 (18.8%)
Lower mediastinum	6/74 (8.1%)	3/20 (15.0%)	6/16 (37.5%)

EGJ, esophagogastric junction; MLN, mediastinal lymph node.

We used multivariate analysis to identify the risk factors for MLN metastasis. The location of the tumor epicenter (HR; 1.131 [1.018‐1.256], *P* = 0.022) and depth of tumor invasion (HR; 0.354 [0.174‐0.719], *P* = 0.004) were among the significant risks for MLN metastasis/recurrence.

### 3.5 Validation of the algorithm

The purpose of the additional test was to verify the validity of the algorithm. We included an additional 22 patients treated between January 2014 and November 2016. Our algorithm was developed based on results obtained from an earlier study of 110 cases treated between 2000 and 2013. The additional 22 cases were divided into two groups (selected/non‐selected groups) using the algorithm, and the MLN metastasis/recurrence rates were then compared. The algorithm was not used to choose the surgical procedure, but rather as a validation check. In the additional study, 11 patients were treated by the transhiatal approach and 11 patients were treated by the transthoracic approach. Seven patients were placed in the selected group that showed significantly higher mediastinal metastasis/recurrence rates (selected group vs non‐selected group: upper/middle MLN; 42.9% vs 4.2%, lower MLN; 42.9% vs 0%, total MLN; 57.1% vs 6.7%).

## 4 DISCUSSION

**Figure 2:**
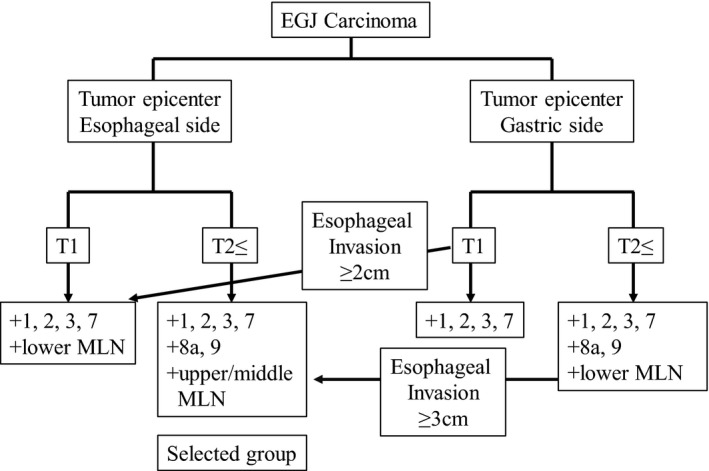
Treatment algorithm for esophagogastric junction (EGJ) carcinoma. We created a surgical algorithm for EGJ carcinoma including adenocarcinoma and squamous cell carcinoma that consisted of three important factors: location of the tumor epicenter, T factor, and degree of esophageal invasion. MLN, mediastinal lymph node

In the present study, we created a surgical algorithm for the treatment of EGJ carcinoma, including AC and SCC, which includes three important factors: location of the tumor epicenter, T factor status, and length of esophageal invasion (Figure 2). Tumor location and T‐factor status were the first two steps in this algorithm as multivariate analysis showed that tumor center location and pT status were related to MLN metastasis. Our algorithm suggests that patients in whom the tumor epicenter is located above the EGJ, and who are also of T1 status, should undergo lower MLN dissection by the transhiatal approach. We included 15 patients with pT1 tumors in whom the tumor epicenter was located above the EGJ; two of these patients (13.3%) had lower MLN metastasis. For advanced (≥T2 status) cases, upper and middle MLN dissection using the transthoracic approach is recommended. In the current study, upper and middle MLN metastasis was detected in 16.7% of the patients who underwent upper MLN dissection (5/30), and the therapeutic index was 8.9 (relatively high). Therefore, upper and middle MLN dissection by the transthoracic approach was therapeutically valuable in patients at high risk of upper and middle MLN metastasis.

For patients in whom the tumor epicenter is located below the EGJ, MLN dissection is generally not necessary for those of T1 status. In the current study, the 22 patients with pT1 tumors whose epicenters were located on the gastric side did not show MLN metastasis or recurrence. However, when the length of esophageal invasion is ≥2 cm, lower MLN dissection should be considered because the MLN metastasis/recurrence rate increases when the length of esophageal invasion exceeds 2 cm (Table 5). For advanced cases, lower MLN dissection should be carried out because the lower MLN metastasis/recurrence rate was 13.5% (5/37) and the therapeutic index of the lower MLN (#110) was high (12.2). In addition, upper and middle MLN dissection should be considered for advanced cases showing extensive esophageal invasion (≥3 cm). Four patients in whom the tumor epicenter was located below the EGJ showed upper and middle MLN metastasis/recurrence, including one case lacking any other recurrent lesions. This suggests that prophylactic MLN dissection by the transthoracic approach prevented recurrence in at least one case. In addition, when the length of esophageal invasion was ≥3 cm, the upper and middle MLN metastasis/recurrence rate increased to 18.8% (Table 5). In a previous report, the length of esophageal invasion was also related to MLN metastasis.^20^

In the present study, we selected patients at high risk of upper or middle MLN metastasis according to the following criteria: (i) advanced (≥T2 status) patients in whom the tumor epicenter was located above the EGJ; and (ii) advanced patients in whom the tumor epicenter was located below the EGJ but who showed esophageal invasion ≥3 cm. Rates of upper and middle MLN metastasis/recurrence in these two groups of patients were 17.9% and 15.4%, respectively, and the total MLN metastasis/recurrence rate was 43.6% (Table 6).

**Table 6:** Mediastinal lymph node metastasis rate in patients in the selected and non‐selected groups

	Selected group N = 39	Non‐selected group N = 71	*P*‐value
Upper MLN	7 (17.9%)	3 (4.2%)	0.022
Middle MLN	6 (15.4%)	1 (1.4%)	0.008
Lower MLN	12 (30.8%)	3 (4.2%)	<0.001
Total MLN	17 (43.6%)	6 (8.5%)	<0.001

MLN, mediastinal lymph node. Selected group (≥T2 and tumor epicenter located above the esophagogastric junction or below the esophagogastric junction with ≥3 cm esophageal invasion).

To validate our algorithm, we additionally surveyed 22 EGJ carcinoma patients treated from January 2014 to November 2016, of whom seven (31.8%) were classified as the selected group and 15 were classified as the non‐selected group. Patients of the selected group showed a higher rate of metastasis/recurrence in the mediastinum lymph node (upper and middle MLN 42.9% [3/7]; lower MLN 42.9% [3/7]; total MLN 57.1% [4/7]) as compared to those of the non‐selected group (upper and middle MLN 6.7% [1/15]; lower MLN 0% [0/15]; total MLN 6.7% [1/15]).

A major prospective Dutch trial compared right thoracic and transhiatal approaches for the treatment of Siewert type I and II tumors. Although the survival rates of patients with Siewert type II tumors did not differ between the groups, a subgroup analysis of patients with nodal metastases showed a significantly better survival following treatment by the transthoracic rather than the transhiatal approach. Tumor epicenters for EGJ carcinomas as defined by Nishi's classification are 1 cm higher (2 cm above and 2 cm below the EGJ) than those of Siewert type II carcinomas (1 cm above and 2 cm below the EGJ). Thus, the subgroup analysis suggested that MLN dissection using the transthoracic approach is effective in patients with EGJ carcinomas with nodal metastasis as defined by Nishi's classification.

In terms of the preoperative diagnostic accuracies of the three elements of our algorithm, we first address the location of the tumor epicenter. In five patients ultimately diagnosed with esophageal‐side tumors, location of the tumor epicenters was preoperatively judged as the gastric side. However, in these cases, the tumor epicenters were located just above (0 mm from) the EGJ. Therefore, these are not actually errors, as they are related to how to define a tumor on the EGJ (esophageal or gastric). In another six (5.5%) cases, the patients were ultimately diagnosed with gastric‐side tumors, although the location of the tumor epicenters was preoperatively judged as the esophageal side. Five tumors had maximum diameters >4 cm, rendering endoscopic identification of the tumor epicenters difficult. Endoscopic diagnosis of the tumor epicenters of large lesions requires further work.

Second, in terms of the preoperative diagnostic accuracy of T1 tumors, of 36 cases diagnosed preoperatively, 32 (89%) were T1 and four were T2 on pathological diagnosis. T1 cases were thus not misdiagnosed; since April 2007, problems with the depth of vision seem to be decreasing because of improvements in endoscopic instruments. If the four T2 cases had been correctly diagnosed before surgery, lower MLN dissection would have been considered, but none of the four cases developed recurrence. However, it should be noted that these four cases were not obviously T2 tumor endoscopically. As the first two steps of our algorithm (location of the tumor epicenter and early/advanced carcinoma) are simple, the effect of the errors on surgical decision‐making was small.

Third, in terms of the preoperative length of esophageal invasion, the precise length (in cm) was not noted prior to surgery in some cases, especially older cases; the correctness (or otherwise) of length evaluation is thus unknown.

In addition, in terms of the preoperative diagnostic accuracy of MLN metastasis, six cases showed CT findings of MLN metastasis prior to surgery, of whom five were ultimately found to have mediastinal metastases. The other 13 cases showed no CT findings of MLN metastasis, but such metastases were discovered on pathological evaluation. CT sensitivity for diagnosis of MLN metastasis was 27.8% (5/18) and the specificity was 98.9% (91/92) (Table S1). This means that patients with enlarged lymph nodes (thus clinically positive) were at high risk of metastasis, but clinically negative cases might nonetheless have metastases. Thus, estimation of the risk of MLN metastasis by reference to tumor location and depth, and the length of esophageal invasion, is necessary.

All deaths except two were caused by the original disease. The two exceptions were deaths caused by postoperative complications (pneumonia). One patient had an AC and the other a SCC. The prognosis did not differ significantly between the SCC and AC groups. However, SCC was associated with a lower 3‐year survival rate, and the survival curve trended downward, reflecting the histological malignancy of SCC. However, if a tumor was present in the same location, the initial lymphatic ducts invaded would be identical and the range of lymph nodes to be dissected would thus be similar regardless of histological type. Indeed, for esophageal carcinoma, treatment (including the extent of lymph node dissection for SCC and AC) does not differ for tumors located in the same area.

Our study had certain limitations. First, the work was retrospective in nature. Second, the procedures were at the discretion of the surgeon; in other words, surgery was not standardized. This means that the effects of MLN dissection are difficult to interpret. Compared with evaluation of lymph nodes #1 and #3 (which were dissected in all cases), therapeutic evaluation of MLN dissection was less reliable, but the MLN therapeutic index was not negligible. In addition, when we included cases showing initial MLN recurrence, we found that recurrence could be prevented by MLN dissection. Of course, the possibility that recurrence cannot be prevented must also be considered. In the future, further investigation of a greater number of cases undergoing upper and middle MLN dissection by reference to our algorithm is required. Third, the median follow‐up period was 51.5 months (>3 years), but some patients with a short observation period (lost to follow up) were included. In total, 18 patients were lost to follow up over 36 months (12 cases, follow up less than 1 year; 1 case, less than 2 years; 5 cases, less than 3 years).

In conclusion, location of the tumor epicenter, depth of invasion (T factor), and extent of esophageal invasion should be considered when choosing a surgical strategy to treat EGJ carcinoma. Our algorithm including these factors was effective in identifying patients at high risk of MLN metastasis. The therapeutic utility of MLN dissection to treat EGJ carcinomas was relatively high in patients showing MLN metastasis.

## DISCLOSURE

Conflicts of Interest: Authors declare no conflicts of interest for this article.

This study was approved by Keio University School of Medicine an Ethical Committee (No: 20150044).
