# Supplementary material for: High‐risk group of upper and middle mediastinal lymph node metastasis in patients with esophagogastric junction carcinoma
Source: Ann Gastroenterol Surg. 2018 Oct 13;2(6):419–27. doi: 10.1002/ags3.12215 (PMC6236104; doi:10.1002/ags3.12215)
Supplement: Supplementary file 1 [file AGS3-2-419-s001.docx]

| **Supporting table1.**  Sensitivity and specificity of mediastinal lymph node metastasis diagnosis on CT scan. | | | |
| --- | --- | --- | --- |
|  | Pathological  MLN (+) | Pathological  MLN (-) | Total |
| Clinical MLN (+) | 5 | 1 | 6 |
| Clinical MLN (-) | 13 | 91 | 104 |
| Total | 18 | 92 | 110 |
| Sensitivity: 27.8% (5/18), Specificity: 98.9% (91/92) | | | |
